# Time Trend Analysis of Cancer‏ Incidence in Caspian Sea, 2004 – 2009: A Population-based Cancer Registries Study (northern Iran)

**Published:** 2016

**Authors:** Hamid Salehiniya, Sakineh Ghobadi Dashdebi, Hosein Rafiemanesh, Abdollah Mohammadian-Hafshejani, Mostafa Enayatrad

**Affiliations:** 1 Minimally Invasive Surgery Research Center, Iran University of Medical Sciences, Tehran, Iran.; 2 Department of Epidemiology and Biostatistics, School of Public Health, Tehran University of Medical Sciences, Tehran, Iran.; 3 Department of Epidemiology and Biostatistics, School of Public Health, Urmia University of Medical Sciences, Urmia, Iran.; 4 Department of Epidemiology and Biostatistics, School of Public Health, Tehran University of Medical Sciences, Tehran, Iran.; 5 Department of Epidemiology and Biostatistics, School of Public Health, Shahid Beheshti University of Medical Sciences, Tehran, Iran.

**Keywords:** Epidemiology, Trend, Cancer, Caspian Sea, Iran.

## Abstract

**Background:**

Cancer is a major public health problem in the world. In Iran especially after a transition to a dynamic and urban community, the pattern of cancer has changed significantly. An important change occurred regarding the incidence of cancer at the southern shores of the Caspian Sea, including Gilan, Mazandaran and Golestan province. This study was designed it investigate the epidemiology and changes in trend of cancer incidence in the geographic region of the Caspian Sea (North of Iran).

**Methods:**

Data were collected from Cancer Registry Center report of Iran health deputy. Trends of incidence were analyzed by joinpoint regression analysis.

**Results:**

During the study period year (2004-2009), 33,807 cases of cancer had been recorded in three provinces of Gilan, Mazandran and Golstan. Joinpoint analysis indicated a significant increase in age-standardized incidence rates (ASR) with an average annual percentage change (AAPC) 10.3, 8.5 and 5.2 in Gilan, Mazandaran and Golestan, respectively. The most common cancer in these provinces were correspondingly cancer of stomach, breast, skin, colorectal and bladder, respectively.

**Conclusion:**

The incidence of cancer tends to be increasing in North of Iran. These findings warrant the epidemiologic studies are helpful in planning preventive programs and recognition of risk factors.

Cancer is a major public health problem and one of the world’s leading causes of death (1-3). Cancer occurs in all age groups but with variability prevalence in different geographic regions (4). According to the World Health Organization (WHO), 7.6 million death reports were recorded in 2005 that increased to 8.2 million people in 2012 (5). Epidemiological pattern of cancer is different in the developed and developing countries from different perspectives (6). While the pattern in the developed coutries is decreasing but in the developing countries is rising (7). Currently, cancer is the first cause of death in the developed countries and the second one in the developing countries (3, 7-10). In Iran, cancer is the third cause of death with the annual incidence of 51,000 new cases(11, 12) after cardiovascular diseases and accidents (2, 13) considering the demographic and epidemiological changes and increasing process of risk factors and increasing life expectancy and the number of elderly in Iran, it is expected that the rate of cancer may increase rapidly in the following decades especially in the developing countries, including Iran(5, 6, 13-15).

So, 20 million new cases is expected by 2025 and has been supposed that the rate of new cases reaches from 56% in 2008 to 60% in 2030 (5, 16). In Iran especially after transition into a dynamic and urban community important changes have taken place on patterns of this illness (17-19). In spite of the limitations in the pathological diagnosis of some types of cancers such as liver, pancreas, lung, ovarian, retinoblastoma and the central nervous system, and considering this fact that there is not any national screening program for cancers such as prostate, colorectal and breast, the actual and expected number of cancers cases is higher than the reported values. However, according to the first national report on the occurrence and death of cancer published in 2009, it has recorded 55,855 cancer cases from March 2005 to March 2006, and 3027 new cases of cancer are available by the national program (13). The provinces in the southern shores of the Caspian Sea, including Gilan, Mazandaran and Golestan an area over 66/58250 km^2^, have a population of about 7,331,831(20). Despite the improvement in standards of living in these regions after 1979, the first population-based study was conducted in the Caspian Sea in 2003. The results compared with the reports of the last 30 years indicated a significant change in the occurrence of cancer in these regions , so that esophageal cancer incidence rate has reduced to fewer than half the rate reported 30 years ago, although the incidence rates of colorectal and breast cancers have increased significantly (21). 

In another study in 2010 in the north of the country, age-specific rate of cancer for all kinds of cancer in men has been estimated at 132-156 in 100,000 men and 96-136 at 100,000 for women (12). According to geographical differences, racial and different habits of people in each area (10) resulted in different rates of cancer incidence in various regions of Iran (22), The prevalence and rates of awareness and the causes of changes in pattern of cancer incidence is heplful for preventive planning programs (1, 16). 

This study aimed at investigating the epidemiology and changes in the pattern and trend of cancer incidence in the southern shore of the Caspian Sea (north of Iran).

## Methods

**Figure1:**
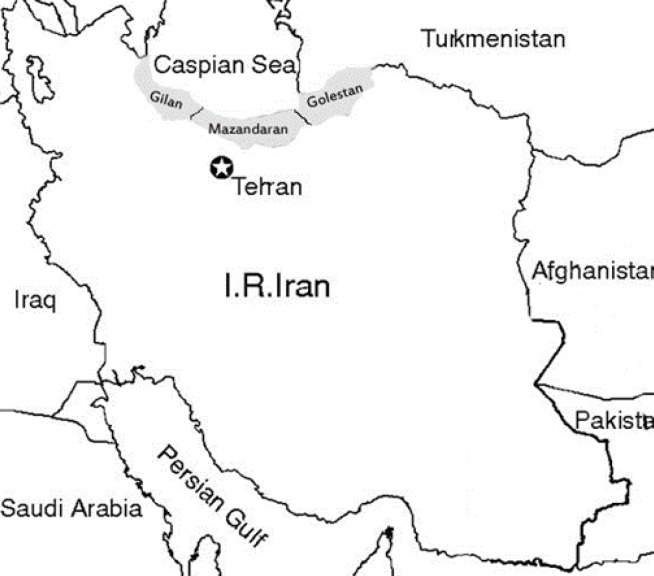
Geographic situation of Gilan, Mazandaran and Golestan, Iran

**Data source:** This cross- sectional study was performed in Gilan, Mazandaran and Golestan Province in Iran. These three provinces are located in the geographic region of southern shores of the Caspian Sea in North of Iran (figure1). Data were collected retrospectively by reviewing all medical records of cancer patients registered in Cancer Registry Center of health deputy for Gilan, Mazandaran and Golestan provinces during a 6-year period (2004-2009) (23). The date of diagnosis was confirmed coded and was based on the International Classification of Diseases for Oncology (ICD-O).

**Statistical analysis:** Age-standardized rates of cancer incidence were calculated by the direct standardization method, using the world standard population as a reference (24). To describe incidence time trends, we carried out joinpoint regression analysis using the software joinpoint regression program, Version 4.1.1.1 October 2014 (25). The analysis included logarithmic transformation of the rates. The aim of the approach is to identify possible joinpoints where a significant change in the trend occurs. The method identifies joinpoints based on regression models with 0-1 joinpoints. In this study, 0 joinpiont (full model) was a significant model. The final model selected was the most parsimonious, with the estimated annual percent change (APC) based on the trend within each segment (26). All statistical tests were two sided.

## Results

During the study years 2004-2009 33, 807 cases of cancer had been recorded in three provinces. Among these, 55.78% patients were men and 44.22% were women. The total number of recorded cases were 12,399 and 6,177 and 15, 231patients were recorded for Gilan, Mazandaran and Golestan respectively. The standardized average age in six studies was 112.10 (per hundred thousand population), in Mazandaran, 111.96 (per hundred thousand population) in Gilan and 84.99 (per thousand population) Golestan in respectively (table1).

**Table 1:** Age standardized incidence rates (ASR) and number of cases according to sex, years and region, in Caspian Sea province, Iran (2004 – 2009

		**Total**		**Male**		**Female**	
**Province**	**year**	**Count**	**ASR**	**Count**	**ASR**	**Count**	**ASR**
Gilan	2004	1575	73.22	917	85.13	658	61.31
	2005	2045	111.48	1157	118.17	888	104.78
	2006	1877	103.81	1076	112.32	801	95.30
	2007	2142	121.07	1217	130.25	925	111.88
	2008	2436	137.39	1401	149.16	1035	125.61
	2009	2324	124.80	1319	142.68	1005	106.92
Mazandaran	2004	2094	91.40	1196	105.48	898	77.32
	2005	2160	97.73	1248	106.91	912	88.54
	2006	2399	108.75	1321	112.91	1078	104.59
	2007	2218	100.02	1180	100.10	1038	99.93
	2008	3267	143.98	1779	146.28	1488	141.67
	2009	3093	130.73	1676	142.87	1417	118.59
Golestan	2004	757	74.36	409	78.63	348	70.09
	2005	940	72.72	509	75.10	431	70.33
	2006	1099	90.14	625	98.89	474	81.39
	2007	1076	87.70	579	89.95	497	85.44
	2008	1129	93.59	620	97.35	509	89.83
	2009	1176	91.45	627	100.36	549	82.53

Standardized incidence rate in all provinces in the study showed an increasing trend. Joinpoint analysis indicated a significant increase in incidence (ASR) with an average annual percentage change (AAPC), 10.3 in Gilan, 8.5 in Mazandaran and 5.2 in Golestan. AAPC between three studied provinces was greater in Gilan than Mazandaran and Golestan. The study of changes did not show a significant increase using 1 joinpoint in 2007. The changes showed significant increasing procedure in some cases according to gender separation for the full model (2004-2009) (table2).

**Table 2:** Joinpoint analyses of cancers incidence in three Northern provinces (Caspian Sea) in Iran (Gilan, Mazandaran and Golestan); 2004–2009

			**Total**		**Male**		**Female**	
	**Trends**	**APC**	**APPC**	**95% CI**	**APC**	**95% CI**	**APC**	**95% CI**
Gilan	2004–2007	16.6	-	-80.8–608				
	2007–2009	0.5	-	-97.3–3602.5				
	2004–2009	-	10.3*	0.8–20.8	10.3*	3.4–17.7	10.5	-2.4–25
Mazandaran	2004–2007	6.1	-	-8.3–22.3				
	2007–2009	12.8	-	-15.8–51.1				
	2004–2009	-	8.5*	1.4–16.2	6.9	-1.2–15.6	10.5*	2.4–19.3
Golestan	2004–2006	10.2	-	-42.3–110.4				
	2006–2009	2.3	-	-26–41.3				
	2004–2009	-	5.2*	0.3–10.8	5.6*	0.1–11.4	4.7*	0.3–9.2

* .APC (Annual Percentage Change) and AAPC (Average Annual Percentage Change) is significantly different from zero at alpha = 0.05

**Figure 2:**
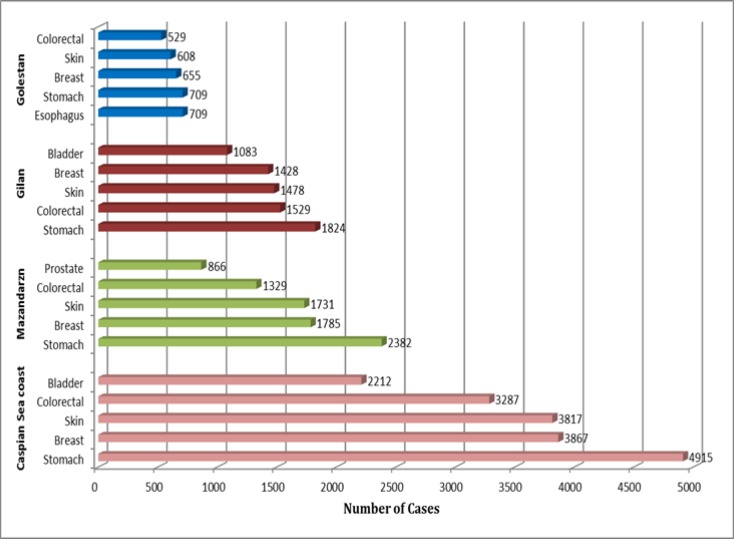
Five common cancers along the provinces, of Gilan, Mazandaran and Golestan according to sum of number cases (2004 – 2009).

Based on the average standardized incidence rate (per 100 thousand), the most common cancer among the women living in Mazandaran over the study were breast (25.96), skin (12.17), stomach (11.00), colorectal (9.94), esophagus (8.31), respectively uhile for the men: stomach (24.44), skin (15.15), prostate (1163), colorectal (10.46) and bladder (9.76), In Gilan, the most common cancers among women were; breast cancer (26.09), colorectal (13.14), skin (11.67), stomach (9.71) and esophagus (5.61) and for the men: stomach (23.12), skin (16.13), bladder (15.41), colorectal (13:50) and esophagus (7.47). In Golestan the most vommon cancers for women were: breast (17.27), esophagus (10:42), colorectal (7.20), skin (6.84) and stomach, respectively (6.46) and stomach (16:09), esophagus (12:31), skin (10.94), colorectal (7.83) and bladder for the men (6.77). Based on the total reported cases during the six years of the study, the most common cancers in both genders in Mazandaran were Stomach cancer, breast, skin, colorectal, and prostate, stomach, skin, breast, colorectal and bladder, in Gilan; esophagus, stomach, breast, skin , and colorectal in Golestan. In total, the most common cancers in the beaches of the Caspian Sea (Gilan, Mazandaran and Golestan) were stomach, breast, skin, colorectal and bladder, respectively (figure 2).

## Discussion

During the years 2004-2009 in the provinces of Gilan, Mazandaran and Golestan, North of Iran, total of 33, 807 cases of cancer had been recorded, 55.78% and 44.22% in men and women, respectively. Standardized incidence rate showed a trend an increase. Joinpoint analysis indicated a significant increase in ASR with an average annual percentage change (AAPC), 10.3 and 5.2 in Gilan, Mazandaran and Golestan, respectively. The five most common cancers in these geographic regions (Gilan, Mazandaran and Golestan) were stomach, breast, skin, colorectal and bladder, respectively. Epidemiological pattern of cancer incidence is different in the developing countries such as Iran compared to developed countries (6). The correct understanding of the patterns and the determination of changes trend of cancer in different regions of the country will help to discover the causes of changes and policies in encountering with a “tsunami” called cancer. The study of changing trends in cancer during years 2004 to 2008 in Khuzestan shows 2669 new cases of cancer (53% men, 47% women). The age-standardized incidence rates were 153.10 and 156.10 for men and women, respectively (1). Based on the results of this study 33,807 cases, have been recorded (55.78% men and 44.22% women) for the three provinces. The number of total reported cases by each provinces included 12,399 cases for Gilan, 15,231 cases in Mazandaran and 6177 cases in Golestan. The age-standardized incidence of cancer in the country was 98 and 110 per 100,000 population per year for men and women, respectively (13). 

According to a population-based study in the north, the incidence in men and women has been reported 132 -156 and 96-136 per 100,000 population per year. Semnani et al. in northern geographic region of Iran found a rate of 134.7 and 104.5 per 100,000 population per year for men and women, respectively (21). According to this study, the highest age-standardized incidence rate during six years of study is in Mazandaran was (112.10 per 100,000 population per year), in Gilan (111.96 per 100,000 population per year), and finally in Golestan (84.99 per 100,000 population per year). The differences in the age-standardized incidence rate may be attributed to methods of diagnosing the type of cancer. However, the age-standardized incidence rates show an increasing trend in all studied provinces like Khuzestan (1) and the whole country (13). 

While in America the overall incidence of cancer in both men and women decreased. The reason of its decline can be attributed mainly to reduction in cancer in men (lung, prostate and colorectal) and a decrease of leading cancers among women (breast and colorectal) (3). The most reported cancers among women and men population were breast cancer and stomach cancer, respectively (13, 17). 

The results of a study by Yazdi Zadeh et al. show that gastric cancer has increased slowly, whereas, the cancer of colon has increased rapidly, during thirty years of research (17). The rapid increase in colon cancer is similar to the trend in many other countries (17). Also, in a study in Gilan confirmed a similar results (2). In spite of the improvement in the standards of living since 1979 in the study regions, the incidence of colorectal cancer and breast cancer have increased (21). Breast cancer in Golestan is the biggest kind of cancer among women and the third common kind of cancer in the population (8). It is seen that Colon cancer is similar all over the country and also one of the main problems in many other parts of the world. Based on the results of this study, the most prevalent kinds of cancer for women in Gilan are breast, colorectal, skin, stomach, esophagus, and in the male population, are respectively, stomach, skin, bladder, colorectal, esophagus. In Mazandaran, the most common kinds of cancer in the female population are breast, skin, stomach, colorectal and esophagus, and in men are stomach, skin, prostate, colorectal and bladder. 

Finally, this report is as follows: In Golestan, female population has shown breast, esophageal, colorectal, skin, stomach cancers and among men stomach, esophagus, skin, colorectal and bladder. However, bladder cancer is lower in Asian countries than Western countries but it is one of the most common kinds of cancer in Iran (6) and the fifth most common kind of cancer among men in North of Iran. It is worth noting that esophageal cancer is of high prevalence in the population of women and men in the Northern provinces, especially in Golestan. While, the age-standardized incidence rate of this cancer is 2 and 3 for men and women, respectively in Kerman. While it has been estimated 43 and 36 in Golestan (12). The differences had been of great consideration in rates of cancer in Golestan, compared to other parts of the country during the last 35 years and several studies were performed in this context. Higher rate of cancer in these regions has been attributed to several factors including insufficient consumption of fruits and vegetables, drinking hot tea, tobacco use, Helicobacter pylori infection in the stomach, drinking contaminated water and genetic predisposition (12). Based on the six-year study, gastric cancer was recorded as the most common kind of cancer in the Caspian Sea. The high rate of stomach cancer has been attributed to the prevalence of H. pylori and use of salted foods and foods containing nitrogen, in countries that are faced to this problem (17). 

Since, the cancer registry system in Iran is still not fully and equally established in all areas yet and sometimes the differences in the quality and coverage of data an observed. Registry of cancer cause was limited to the pathology system hence a large number of cancers were missed. A part of the trend of cancer may be due to improvements in registration and reporting systems during study periods.

In conclusion, the incidence trend of cancer tends in Caspian Sea region provinces. The overall the results of this study showed that the most common cancers in the Caspian Sea region were stomach, skin, colorectal and bladder. 

## Notes

**Funding:** The study was not supported financially.
